# 

**Published:** 1884-11

**Authors:** 


### CONTENTS.

ORIGINAL COMMUNICATIONS: PAGE.

I. Fatal Pelvic Hemorrhage Following the Use of the Aspirator Needle. By A. Reeves Jackson, M.D., Professor of Gynecology in the College of Physicians and Surgeons of Chicago, etc. .... 385

II. Introductory Lecture before the Classes of the Chicago Medical College, Sept. 23, 1884. By J. E. Owen, M.D., Professor of Operative Surgery and Surgical Anatomy, Chicago Medical College, Medical Department Northwestern University, Chicago .... 388

EDITORIAL:

The German Medical Student and Physical Diagnosis .... 414
A Collection of Curiosities .... 419
Literary Note .... 422

BOOK REVIEWS:

Diseases of the Throat and Nose, Including the Pharynx, Larynx, Trachea, Œsophagus, Nose, and Naso-Pharynx. By Morell Mackenzie, M.D. .... 424
Lectures on the Principles and Practice of Medicine. Delivered in Chicago Medical College, Medical Department of the Northwestern University, by Nathan Smith Davis, A.M., M.D., LL.D. .... 425

PAGE.

The National Dispensatory. Containing the Natural History, Chemistry, Pharmacy, Actions and Uses of Medicine. By Alfred Stille, M.D., LL.D., and John M. Maisch, Phar. D. .... 428
The Field of Disease: A Book of Preventive Medicine. By Benjamin Ward Richardson, M.D., LL.D., F.R.S. .... 428
Legal Medicine, Vol. II. Legitimacy, Abortion, Rape, Sodomy, Infanticide, Drowning, Hanging, Suffocation. By Ch. Meynott Tidy .... 431
Illustrations of the Influence of the Mind upon the Body in Health and Disease, Designed to Elucidate the Action of the Imagination. By Daniel Hack Tuke, M.D., LL.D. .... 432
A Treatise on Bright's Disease of the Kidneys, its Pathology, Diagnosis and Treatment. With Chapters on the Anatomy of the Kidney, Albuminuria, and Urinary Secretions. By Henry B. Millard, M.D., A.M. .... 443

SOCIETY PROCEEDINGS:

Chicago Medical Society .... 435
Chicago Pathological Society .... 458
A Medical Department in the Chicago Medical Library .... 467
The Floating Hospital Association .... 468

CORRESPONDENCE:

Letters from Italy .... 470

